# Heart Failure in Patients with Chronic Kidney Disease: A Systematic Integrative Review

**DOI:** 10.1155/2014/937398

**Published:** 2014-05-15

**Authors:** Liviu Segall, Ionut Nistor, Adrian Covic

**Affiliations:** Nephrology Department, Faculty of Medicine, University of Medicine and Pharmacy “Gr. T. Popa”, Strada. Universităţii No. 16, 700115 Iaşi, Romania

## Abstract

*Introduction*. Heart failure (HF) is highly prevalent in patients with chronic kidney disease (CKD) and end-stage renal disease (ESRD) and is strongly associated with mortality in these patients. However, the treatment of HF in this population is largely unclear. * Study Design*. We conducted a systematic integrative review of the literature to assess the current evidence of HF treatment in CKD patients, searching electronic databases in April 2014. Synthesis used narrative methods. * Setting and Population*. We focused on adults with a primary diagnosis of CKD and HF. * Selection Criteria for Studies*. We included studies of any design, quantitative or qualitative. * Interventions*. HF treatment was defined as any formal means taken to improve the symptoms of HF and/or the heart structure and function abnormalities. * Outcomes*. Measures of all kinds were considered of interest. * Results.* Of 1,439 results returned by database searches, 79 articles met inclusion criteria. A further 23 relevant articles were identified by hand searching. * Conclusions.* Control of fluid overload, the use of beta-blockers and angiotensin-converting enzyme inhibitors or angiotensin receptor blockers, and optimization of dialysis appear to be the most important methods to treat HF in CKD and ESRD patients. Aldosterone antagonists and digitalis glycosides may additionally be considered; however, their use is associated with significant risks. The role of anemia correction, control of CKD-mineral and bone disorder, and cardiac resynchronization therapy are also discussed.

## 1. Epidemiology

During the past decade, the worldwide medical community has become increasingly aware of the fact that chronic kidney disease (CKD) is a strong and independent risk factor for cardiovascular disease (CVD). In the US, for example, the prevalence of CVD in CKD patients reaches 63%, in contrast with only 5.8% in people without CKD, and this prevalence is directly correlated with the severity of CKD [1]. In dialysis-dependent end-stage renal disease (ESRD) patients, the risk of cardiovascular (CV) mortality is 10-fold to 20-fold higher than in age- and gender-matched control subjects without CKD [2, 3]. This remarkable association of CKD with CVD is commonly explained by a typical clustering of several CV risk factors in patients with CKD; these factors may be classified as “traditional” (including advanced age, hypertension, diabetes, and dyslipidemia) and “nontraditional” (CKD-specific) ones (such as anemia, volume overload, mineral metabolism abnormalities, proteinuria, malnutrition, oxidative stress, and inflammation).

Heart failure (HF) is the leading CV complication in CKD patients and its prevalence increases with declining kidney function [4]. In the Atherosclerosis Risk in Communities (ARIC) study [5], a large, population-based study of US adults, the incidence of HF was 3-fold higher in individuals with an estimated glomerular filtration rate (GFR) <60 mL/min/1.73 m^2^, compared with the reference group with an estimated GFR ≥90 mL/min/1.73 m^2^. In dialysis patients, the presence of HF at the start of dialysis is a strong and independent predictor of short-term [6] and long-term mortality, in both hemodialysis (HD) [7] and peritoneal dialysis (PD) patients [8]. The median survival of dialysis patients with baseline HF has been estimated to be 36 months, in contrast with 62 months for those without baseline HF [7]. Over 80% of ESRD patients who are recently diagnosed with HF are expected to die within only three years from the time of this diagnosis [9].

## 2. Pathophysiology

Abnormalities of left ventricular (LV) structure and function are very common in CKD and ESRD patients. Among ESRD patients, approximately 73.4% of those who are started on dialysis have LVH, 35.8% have LV dilatation, and 14.8% have LV systolic dysfunction [10]. Usually, LVH does not regress or even aggravates with time on dialysis and its presence is associated with a high risk of mortality and CV events, including sudden cardiac death [11].

Myocardial hypertrophy is associated with a reduction in the capillary density [12], which creates an imbalance between oxygen demands and supplies, thus causing ischemia [13]. Ischemia promotes myocardial cell apoptosis, as well as extracellular matrix and collagen accumulation, leading to interstitial fibrosis, which, in turn, induces LV stiffness, increased LV filling pressure, impaired diastolic filling, and diastolic dysfunction [14, 15]. Moreover, myocardial fibrosis aggravates ischemia, by reduction of capillary density and coronary reserve [16], and considerably increases the risk of ventricular arrhythmias and sudden cardiac death [17–19]. Associated coronary artery disease—also, very common in patients with CKD and ESRD—further contributes to ischemia, myocardial cell damage, and fibrosis [11].

From a hemodynamic view, LVH is an adaptive remodeling process of the LV, which compensates the increase in cardiac work induced by an increased afterload (pressure overload), an increased preload (volume overload), or both. Increased afterload may result from arterial hypertension, arterial stiffness, or valvular aortic stenosis and typically leads to a concentric thickening of the LV wall (concentric hypertrophy), which is meant to boost the intraventricular systolic pressure. Increased preload may be due to hypervolemia, anemia, and (in HD patients) high blood flow arteriovenous fistula; volume overload leads to the development of LV dilatation (eccentric LVH), by accumulation of new myocardial sarcomeres in series [20]. Afterload and preload factors often coexist in various degrees and combinations, with an additive or synergistic effect, which explains why both patterns, as well as a mixed pattern of LVH, are commonly seen in CKD patients [21].

A large number of nonhemodynamic factors also contribute to the development of LVH and cardiomyopathy in CKD patients [12]. For example, hyperphosphatemia has been associated with high blood pressure (BP) [22], increased LV mass [23, 24], and diastolic dysfunction [25]. Excess angiotensin II can accumulate in the heart and promote myocyte hypertrophy, interstitial fibrosis, and microvascular disease, as well as cardiac conduction disturbances, QT prolongation, and arrhythmias [26]. High serum aldosterone, resulting from activation of renin-angiotensin system or other pathways, can induce myocardial fibrosis, possibly by release of transforming growth factor *β* [17, 27]. Sympathetic overactivity, which has been demonstrated in CKD, is also deleterious to the heart and may induce LV concentric remodeling [28].

## 3. Diagnosis

HF is defined as a syndrome that can result from any structural or functional cardiac disorder that impairs the ability of the heart to function as a pump to support a physiological circulation [29]. HF may occur as a result of either systolic or diastolic dysfunction. The definition of HF requires the presence of symptoms, signs, and objective evidence of a structural or functional cardiac abnormality [30].

### 3.1. Cardiac Imaging Studies

Two-dimensional (2D) echocardiography (including Doppler imaging) is a crucial investigation for the assessment of LV structure and function and for the diagnosis of causes of HF, such as LVH, myocardial ischemia, valvular disease, and pericardial effusion or constriction [31]. Echocardiography should provide measurements of ventricular diameters and volumes, wall thickness, chamber geometry, ejection fraction (EF), and regional wall motion abnormalities. In addition, atrial dimensions and/or volumes have to be measured. All valves must be evaluated for anatomic changes, flow abnormalities, and dysfunctions, such as valvular regurgitation. Combined quantification of mitral valve inflow pattern, pulmonary venous inflow pattern, and mitral annular velocity provides important information about the characteristics of LV filling and left atrial (LA) pressure [32]. Echocardiography has a key role in the diagnosis of systolic versus diastolic HF. Systolic HF is usually defined as HF with a LVEF ≤50% [33, 34]. Diastolic HF—also referred to as HF with normal LVEF—requires three diagnostic criteria: (a) presence of signs and symptoms of HF, (b) presence of normal or mildly abnormal LVEF, and (c) evidence of diastolic LV dysfunction [35].

An echocardiogram is warranted in any CKD patient presenting with new cardiac symptoms or events [36]. For ESRD patients, the kidney disease outcomes quality initiative (KDOQI) guidelines recommend echocardiograms to be performed 1–3 months after the start of dialysis and every 3 years thereafter, regardless of symptoms [37]. Other authors even suggest a closer follow-up, with serial examinations every 12 to 18 months [38, 39].

CKD patients with significant LV systolic dysfunction should undergo an evaluation for coronary artery disease. This may include both noninvasive imaging (stress echocardiography, nuclear imaging, or computed tomographic angiography) and invasive imaging tests (coronary angiography), as recommended by KDOQI guidelines [37]. Coronary angiography is indicated in patients presenting with HF and known or suspected coronary artery disease, except for those ineligible for revascularization therapy. Instead, computed tomographic angiography may be used in cases with an intermediate likelihood of coronary artery disease; however, this procedure has potential technical limitations in CKD, because of the severity of vascular calcifications, which may reduce the accuracy of coronary imaging [31]. Cardiac magnetic resonance imaging (MRI) enables accurate measurements of LV and LA volume and can identify myocardial viability and scar tissue [40]; however, given the issues of cost and availability, as well as the risk of nephrogenic systemic fibrosis from gadolinium contrast in CKD patients, MRI is not currently recommended as a routine imaging test in this population [31].

### 3.2. Assessment of Fluid Status

Assessment of fluid status is very important in all CKD and, particularly, ESRD patients. In those with CKD and HF, volume overload may be both a result and a precipitating factor of the latter condition.

However, the best way to assess fluid status and dry weight in dialysis patients is still an unsolved issue. The ideal method should be highly sensitive and specific, readily available, inexpensive, fast and easy to use by clinicians, and capable of predicting clinical outcomes. Such a method still does not exist. Dry weight in ESRD patients is currently determined in most dialysis centers on a clinical basis, and it is commonly defined as the lowest body weight a patient can tolerate without developing intra- or interdialytic hypotension or other symptoms of dehydration [41, 42]. However, clinical findings have insufficient specificity, sensitivity, and objectivity; they are often contradictory and difficult to interpret, cannot detect small changes in hydration status, and cannot accurately predict the target-dry weight. Furthermore, this decades-old strategy has not contributed to reducing the CV mortality in ESRD patients, which is ultimately one of the main long-term goals of renal replacement therapy. Therefore, more objective and more sophisticated techniques of assessing volume status have been proposed, each of these having its own benefits and limitations.

The echocardiographic measurement of inferior vena cava diameter and collapsibility can accurately predict right atrial pressure and volume status in HD patients, and the adjustment of dry weight based on this technique was shown to prevent intradialytic adverse events, to reduce LV mass and LA size, and to improve quality of life [43, 44].

Relative plasma volume monitoring is a safe and inexpensive procedure that monitors relative plasma volume by analyzing blood density and allows automatic feedback control of the ultrafiltration rate that prevents relative plasma volume from reaching a critical level. This technique has decreased intradialytic hypotension [45], and a recent study suggested that it may aid dry weight assessment during HD [46]. However, there are no norms to guide fluid removal by this method and its benefit for clinical outcomes is not known [47, 48].

One of the most promising methods of assessing dry weight that have emerged in recent years is bioelectrical impedance analysis (BIA). This method estimates body composition, including total body water (TBW), extracellular water (ECW), and intracellular water (ICW), by measuring the body's resistance and reactance to electrical current. It has been validated in healthy subjects and various patient populations by isotope dilution and other body composition techniques [49]. The procedure is safe, simple, and relatively inexpensive. Several studies have proved the usefulness of BIA for the evaluation of dry weight in HD patients. Overhydration >15% of ECW as measured by BIA was demonstrated to predict mortality [50]. MacHek et al. showed that the adjustment of fluid status guided by BIA led to significant reductions in systolic BP and antihypertensive medications in overhydrated HD patients and prevention of adverse events in underhydrated ones [51].

More recently, a novel method has been described that detects pulmonary congestion using ultrasound [52]. Although lung water excess was not directly related to hydration status assessed by BIA, lung water after dialysis showed a strong negative association with LVEF and positive association with LA volume and pulmonary pressure, clearly suggesting that chest ultrasonography may be reliable in detecting subclinical pulmonary congestion in dialysis patients.

### 3.3. Natriuretic Peptides

Atrial natriuretic peptide and brain natriuretic peptide (BNP) are produced by atrial and ventricular myocytes in response to an increase in atrial or ventricular diastolic filling pressure and wall distension [53, 54]. Myocardic cells are released in circulation pro-BNP, a precursor that is subsequently cleaved into the biologically active BNP and the biologically inactive NT-pro-BNP. Thus, plasma levels of BNP and NT-pro-BNP reflect LV wall stress [53]. These levels are greatly increased in patients with HF and they are strongly correlated with the severity of LV systolic and diastolic dysfunction [53, 55], as well as with the severity of HF, as assessed by the New York Heart Association (NYHA) classification [56, 57]. BNP and NT-pro-BNP are currently considered as useful biomarkers for the diagnosis and evaluation of HF in the general population [58, 59]. They also have an important prognostic value, as independent predictors of mortality and other cardiac endpoints in patients with HF [60]. A recent systematic review of available data concluded that HF therapy guided by target-natriuretic peptide concentrations is associated with better outcomes [61].

In CKD and ESRD plasma levels of natriuretic peptides are affected by the impaired renal clearance [62]; however, they still maintain a strong relation with LV end-diastolic wall stress. In dialysis patients, plasma natriuretic peptides have shown significant associations with LVH [63], LV systolic and diastolic dysfunction [62, 64–66], and LA dilatation [62, 67]. Furthermore, it has been demonstrated that BNP and NT-pro-BNP can predict the risk of HF in nondialysis CKD [63] and in PD patients [65], respectively. These data clearly suggest the potential interest in the use of natriuretic peptides in the diagnosis and management of HF in CKD and ESRD [68]. In these populations, however, there is still no evidence for the role of natriuretic peptides in the diagnosis of HF (either rule-in or rule-out), for their prognostic value, or for their utility in guiding the treatment of HF. Specific cutoff levels also need to be determined.

## 4. Treatment

In this section, we discuss the therapy of chronic HF in patients with CKD, looking up to the existing evidence in this population, as well as to relevant trials and guidelines in the general population with HF. However, we shall not refer to the prevention and treatment of various causes of HF (such as coronary artery disease or valvular heart disease) or to the management of complications like arrhythmias, sudden cardiac death, acute pulmonary edema, and cardiogenic shock, which are beyond the scope of this review.

### 4.1. Treatment Goals

The treatment of HF in patients with CKD is unclear, as there is very little strong evidence to support any recommendations. Guidelines for the management of HF in the general population may not apply entirely to those with CKD, since such patients (particularly those with severe renal impairment) were quite often excluded from most of the RCTs that served as a rationale for these guidelines. The paucity of specific evidence and recommendations may explain why CKD patients with HF are less likely to receive certain therapies that are commonly used in the general HF population [69]. Wang and Sanderson [69] pointed out that the main objectives of HF therapy in CKD (as well as in non-CKD) patients are the following: (1) to decrease the preload and afterload and to reduce LVH, (2) to treat myocardial ischemia, and (3) to inhibit neurohumoral hyperactivity, especially the sympathetic nervous system and the renin-angiotensin-aldosterone system (RAAS).

### 4.2. Treatment Options: A Systematic Review

#### 4.2.1. Methods

*Eligibility Criteria*. We conducted electronic searches in the Cochrane Central Register of Controlled Trials (Central—issue 4, 2014) and MEDLINE (1966 to April 2014), using search terms relevant to this review without language restriction (Table S1) see Supplementary Material available online at (http://dx.doi.org/10.1155/2014/937398). In addition, we hand-searched journals, conference proceedings, and current awareness alerts, without language restriction. Two reviewers independently screened the search results by title and abstract, and then full text, to identify eligible trials that fulfilled inclusion criteria. We considered all studies design that assessed any of the following interventions for the treatment of adults with heart failure and chronic kidney disease, as defined by the authors.

*Types of Interventions*. We considered comparisons including diuretics, beta-blockers, angiotensin-converting enzyme inhibitors, angiotensin receptor blockers, aldosterone antagonists, and digitalis glycosides. Optimization of dialysis, the role of anemia correction, control of CKD-mineral and bone disorder, and cardiac resynchronization therapy were also included. 

*Types of Outcome Measures*. Primary outcomes were all-cause mortality and CV mortality. Measures of all other kinds were considered of interest. 

#### 4.2.2. Results

**Figure 1:**
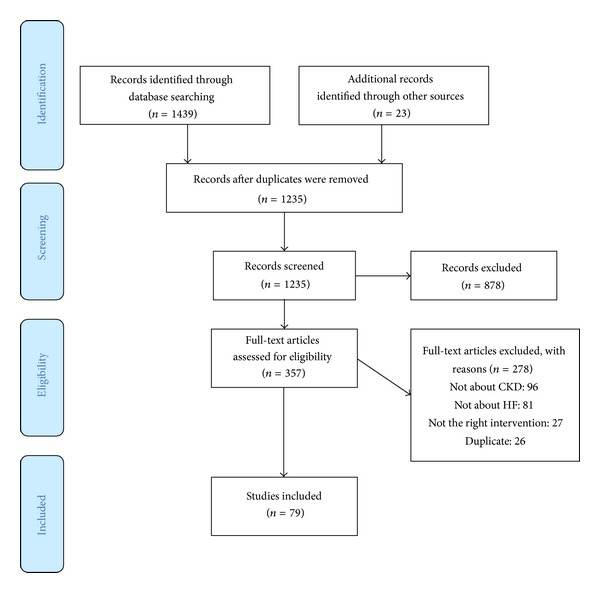
Inclusion and exclusion flow diagram.

*Study Selection*. Of 1,439 results returned by database searches, 79 articles met inclusion criteria. A further 23 relevant articles were identified by hand searching.  Figure 1 shows a summary of inclusion and exclusion algorithm.

#### 4.2.3. Control of Fluid Overload

In CKD patients, maintaining salt and water balance and improving BP control are key strategies to reduce both the risk and the manifestations of HF [70]. The importance of this approach has been demonstrated particularly in dialysis patients, in whom rigorous limitation of salt intake and aggressive ultrafiltration were shown to prevent or reduce LV hypertrophy and dilatation, with little or no antihypertensive medication [71, 72].

Dietary salt restriction is fundamental for CKD patients with HF [12]; however, there are no randomized control trials (RCTs) to confirm its benefit on CV outcomes in this population. Diuretic therapy often requires higher doses than in HF patients with normal kidney function [12]. Loop diuretics should be used as first-line agents in patients with GFR <30 mL/min/1.73 m^2^, because thiazides are relatively ineffective in these cases when used alone [73]. Resistance to loop diuretics is also common in patients with advanced CKD, because of glomerular loss, tubular resistance from chronic diuretic use, secondary hyperaldosteronism, reduced intestinal drug absorption, and inadequate salt and water intake [74]. The diuretic effect can be improved by increasing total daily doses or dosing frequency (to 3 times daily) or by using combination regimens (e.g., furosemide with metolazone or hydrochlorothiazide) [73]. Intravenous bolus administration or continuous infusion of diuretics may be used briefly in resistant cases. Possible side effects, such as hypovolemia, hypokalemia, and hyponatremia, require careful monitoring, particularly when high doses or combination therapy is used [73].

#### 4.2.4. Treatment of Anemia

In non-CKD patients with HF, several studies have reported that treatment of anemia with erythropoiesis-stimulating agents (ESA) can improve functional status, quality of life [75, 76], and LVEF [77]. A meta-analysis of 7 RCTs including 650 patients [78] showed a lower risk of hospitalization for HF, with no difference in adverse events, but also no significant decrease in mortality with rHuEPO treatment.

In patients with CKD and ESRD, anemia has also been associated with LV hypertrophy and dilatation [79]. The presence of anemia during the first year of renal replacement therapy was associated with an increase in the prevalence of LVH [2]. In CKD, a few nonrandomized studies have shown a regression of LVH after correction of anemia [80, 81]; however, prospective RCTs found no evidence that this therapy can improve CV outcomes [81]. An RCT by Foley et al. [82] failed to show regression of LVH after normalization of hemoglobin levels using rHuEPO in HD patients. Several subsequent large RCTs in patients with CKD and ESRD, including the Normal Hematocrit Trial [83], the CREATE (Cardiovascular Risk Reduction by Early Anemia Treatment with Epoetin Beta) study [84], the CHOIR (Correction of Hemoglobin and Outcomes in Renal Insufficiency) study [85], and the TREAT (Trial to Reduce Cardiovascular Events with Aranesp Therapy) study [86], showed an increased risk of death and CV events with erythropoiesis-stimulating agents. Although correction of anemia may improve myocardial oxygen delivery, it is thought that increased blood viscosity and BP associated with rHuEPO therapy could explain the adverse CV effects [87]. Considering these data, target serum hemoglobin of 11 to 12 g/dL has been recommended for CKD patients by the Anaemia Working Group of the European Renal Best Practice [88]. However, no RCT has specifically addressed the optimal hemoglobin level in CKD patients with HF, so far. Therefore, it seems reasonable, for now, to treat these patients similarly to the general CKD population, avoiding the complete correction of anemia [89].

#### 4.2.5. Management of CKD-Mineral and Bone Disorder

Achieving adequate phosphate, calcium, vitamin D, and parathyroid hormone (PTH) levels is a reasonable treatment goal in CKD patients, with or without heart disease, although their benefits for preventing or improving HF in these patients are uncertain.

Hyperphosphatemia can promote LVH, possibly through changes in arterial stiffness, in systemic vascular resistance or through direct myocardial effects [23–25, 90]. However, there are yet no RCTs showing that a therapeutic decrease in serum phosphorus can prevent or reduce LVH or LV dysfunction in CKD patients. The use of non-calcium-containing phosphate binders is preferable, according to KDIGO guidelines [91], although a recent systematic review did not find sufficient evidence to confirm the superiority of these agents over calcium-based binders for CV end-points [92].

Parathyroid hormone has long been regarded as a CV toxin in uremia, particularly on the account of experimental studies. It has recently been suggested that some of the possible cardiac adverse effects of PTH could be mediated by fibroblast growth factor-23 (FGF23), which has been shown to directly induce LVH in both* in vitro* and* in vivo* studies [93, 94]. In ESRD patients, very high PTH levels (e.g., >500 pg/mL) have been associated with persistent LVH [69, 95]. However, there is still no evidence that therapeutic lowering of PTH might prevent or ameliorate HF in CKD patients.

In experimental studies, 25-OH-vitamin D deficiency has been associated with myocardial fibrosis and systolic dysfunction [96]. Low serum vitamin D also stimulates the RAAS, resulting in vasoconstriction and salt and water retention, which further promotes arterial stiffening [97]. In animal models, treatment with active vitamin D inhibits endothelin-induced myocyte hypertrophy [98], reduces LV mass, and improves LV function, in parallel with a decrease in plasma BNP and renin activity [99].

In CKD patients, vitamin D deficiency has also been associated with LV dysfunction and risk of CV events, including HF [100]. Treatment with intravenous calcitriol in a short-term uncontrolled study in HD patients with secondary hyperparathyroidism showed partial regression of LVH and a decrease in plasma renin activity and angiotensin II levels [101]. A very recent randomized study [102] compared the effect of alfacalcidol versus no treatment in 14 patients with CKD stage 4, secondary hyperparathyroidism, and LVH. After 6 months, in the alfacalcidol-treated patients the LV mass index remained stable, but the LV systolic function (shortening fraction) significantly increased, while PTH decreased by 72%. Another recent but uncontrolled study [103] evaluated the effect of cholecalciferol supplementation on cardiac function in 30 HD patients with low vitamin D and low PTH levels. After 6 months, the authors found a significant increase in serum 25(OH)D levels, but no changes in PTH and phosphate, whereas LV mass index was significantly reduced by 16 g/m^2^.

Selective vitamin D receptor activators, such as paricalcitol, provide similar efficacy as vitamin D but are usually not associated with increases in serum concentrations of calcium and phosphorus. This explains the interest in these agents as a potential new approach for CVD in CKD patients [97]. However, the recent PRIMO study [104], a multinational, double-blind, randomized, placebo-controlled trial in 227 patients with stages 3 and 4 CKD, mild-to-moderate LVH, and preserved LVEF, failed to demonstrate any significant effect of paricalcitol on LV mass and diastolic function. Therefore, the role of vitamin D receptor activators in the treatment of cardiomyopathy and HF in CKD patients still remains to be elucidated.

#### 4.2.6. Beta-Blockers

Beta-blockers have been evaluated in more than 20,000 patients with HF in over 20 placebo-controlled clinical trials. These trials have almost unanimously proven that these drugs can alleviate symptoms, improve the NYHA class, increase the LVEF, reduce hospitalizations, and, most importantly, prolong survival. The benefits of beta-blockers have been confirmed in patients with or without coronary artery disease and with or without diabetes, as well as in women and black patients. Favorable results were also seen in patients already taking ACEIs, which suggests that combined blockade of the two neurohormonal systems may have additive effects [32].

Three beta-blockers have been found to reduce mortality in patients with HF: bisoprolol and sustained-release metoprolol, which selectively block beta-1-receptors, and carvedilol, which blocks alpha-1-, beta-1-, and beta-2-receptors. According to the guidelines of the American College of Cardiology Foundation/American Heart Association, a beta-blocker (one of these three agents) should be prescribed to all patients with stable HF due to systolic dysfunction, unless contraindicated or not tolerated [32]. Common side effects of beta-blockers include fluid retention and worsening HF at initiation of therapy, fatigue, bradycardia, and hypotension, and prevention of these effects requires careful dosage titration and clinical monitoring.

In patients with CKD, sympathetic overactivity is thought to play an important role in the pathogenesis of hypertension [105] and LVH [106]. High plasma norepinephrine levels predict adverse CV events and mortality in dialysis patients [107]. However, there is still limited evidence about the efficacy and tolerability of beta-blockers in patients with HF and renal dysfunction, since RCTs in HF have most often excluded individuals with CKD [108].

Several observational studies [109–111] have reported promising results. The Cooperative Cardiovascular Project [110] was a nonrandomized observational study using propensity score matching in patients over 65 years who survived a myocardial infarction. In the 2613 participants on beta-blockers, a greater benefit was noted for patients with serum creatinine levels >2.0 mg/dL. In a retrospective cohort study of 2550 patients enrolled in the US Renal Data System (USRDS) Wave 2 [111], beta-blocker use was associated with a lower adjusted risk of HF and cardiac death in patients without a history of HF; however, no association was observed in those with previous HF.

In predialysis CKD patients, more convincing evidence has been provided by recent* post hoc* analyses of 3 RCTs using beta-blockers in patients with HF [112–114]. The Metoprolol CR/XL Controlled Randomized Intervention Trial in Chronic HF (MERIT-HF) studied the effect of metoprolol in comparison with placebo in 3991 patients with symptomatic systolic HF (NYHA classes II to IV and LVEF < 40%). In a secondary analysis, patients were divided into 3 renal function subgroups: eGFR >60 (*n* = 2496), eGFR 45 to 60 (*n* = 976), and eGFR <45 mL/min/1.73 m^2^ (*n* = 493). The beneficial effect of metoprolol on clinical outcomes was significant and similar across all subgroups. In patients with eGFR <45, beta-blocker treatment was associated with a decrease by almost 60% in total mortality and hospitalizations for HF. Metoprolol was well tolerated in all patients, irrespective of GFR [112]. The cardiac insufficiency bisoprolol study II (CIBIS-II) was a double-blind, randomized comparison of bisoprolol and placebo in 2647 patients with NYHA classes III and IV HF and a LVEF <35%. Renal function impairment, defined as a serum creatinine ≥3.4 mg/dL at baseline, was an exclusion criterion. The trial was stopped prematurely, after a mean follow-up of 1.3 years, as bisoprolol treatment led to a highly significant reduction in all-cause mortality. In a* post hoc* analysis, patients were divided into four subgroups according to baseline eGFR (< 45, 45–60, 60–75, and > 75 mL/min per 1.73 m^2^). The beneficial effects of bisoprolol were shown to be similar among all groups. In those with eGFR <45, the risk of total mortality or hospitalization for HF was significantly decreased by 28%; however, the rate of bisoprolol discontinuation was higher in this subgroup [113]. SENIORS (Study of the Effects of Nebivolol Intervention on Outcomes and Rehospitalization in Seniors with Heart Failure) investigated the effect of nebivolol versus placebo in 2112 elderly patients with HF (age > 70). The primary outcome (composite of all-cause mortality or CV hospital admission) was significantly reduced in those taking nebivolol (31.1% versus 35.3%). When patients were divided by tertiles of eGFR, nebivolol was found to be similarly effective in all groups. In patients with the lowest eGFR (< 55.5 mL/min per 1.73 m^2^), the primary end-point was reduced by 19%, although this did not reach statistical significance. Nebivolol use in these patients was associated with higher rates of drug discontinuation due to bradycardia [114].

There is only one RCT that evaluated the effects of beta-blocker treatment in dialysis patients with HF. In this 12-month study of 114 HD patients with NYHA classes II-III HF and LVEF <30%, carvedilol therapy was associated with a significant improvement in LVEF and NYHA class, compared to placebo [115]. Subsequent 24-month extended follow-up of the same cohort suggested a survival benefit with carvedilol (51.7% mortality rate in the carvedilol group versus 73.2% in the placebo group; *P* < 0.01). There were significantly lower rates of CV mortality (29.3% versus 67.9%; *P* < 0.0001) and all-cause hospital admission (34.5% versus 58.9%; *P* < 0.005) in the carvedilol group than in the placebo group. Secondary end-point analyses showed fewer fatal myocardial infarctions, fatal strokes, and hospital admissions for worsening HF in recipients of carvedilol. A decrease in sudden deaths and pump-failure deaths also was observed, although this did not reach statistical significance. Notably, 2-year echocardiographic data confirmed the significant improvement in LVEF [116].

A recent systematic review and meta-analysis [108] retrieved 6 placebo-controlled trials with beta-blockers, involving 5,972 patients with CKD and systolic HF. The authors found that beta-blocker treatment significantly reduced the risk of all-cause (by 28%) and CV mortality (by 34%), compared to placebo. On the other hand, the risks of bradycardia and hypotension were each increased 5-fold with beta-blocker therapy.

#### 4.2.7. ACE Inhibitors

A subject of many studies, the mechanisms of ACEIs in HF are complex and still not completely understood. By blocking the conversion of angiotensin I to angiotensin II, these drugs promote vasodilation (by reducing the vasoconstrictive effect of angiotensin II) and renal sodium excretion (by decreasing aldosterone release). They inhibit the cardiac RAAS, which is involved in LV hypertrophy and dysfunction. They also block the degradation of bradykinins, thereby stimulating the synthesis of prostaglandins and nitric oxide, which seem to prevent LVH, as well. Other significant effects of ACEIs include the reduction of sympathetic activity, improvement of endothelial function, decrease of proinflammatory cytokines and prothrombotic factors, and stimulation of fibrinolytic factors. All these mechanisms contribute to the amelioration of pulmonary, right ventricular and skeletal muscle function and the increase of arterial compliance [117].

ACEIs have been evaluated in more than 7000 patients with systolic HF, in over 30 placebo-controlled clinical trials. Analyses of these studies showed that these drugs can alleviate symptoms, improve functional status, and reduce the risk of death and hospitalization [30, 32]. These benefits were seen in patients with various severity and causes of HF [32]. US and European guidelines recommend prescription of ACEIs to all patients with HF due to systolic dysfunction (LVEF ≤ 40%), irrespective of symptoms, unless contraindicated or not tolerated [30, 32]. Patients should not be given an ACEI if they have experienced life-threatening adverse reactions (angioedema or anuric renal failure) during previous exposure to the drug or if they are pregnant. They should take an ACEI with caution if they have very low systemic blood pressure (systolic blood pressure less than 80 mm Hg), markedly increased serum levels of creatinine (>3 mg/dL), bilateral renal artery stenosis, or elevated levels of serum potassium (>5.5 mEq/L). Treatment should be initiated at low doses and gradually increased thereafter. The most common adverse effects of ACEIs are hypotension, acute kidney injury, hyperkalemia, and cough [30, 32]. During ACEI therapy, serum creatinine and potassium should be assessed periodically, especially in patients with diabetes and with renal disease [30, 32].

The use of ACEIs in patients with CKD and HF seems reasonable, given the well-established simultaneous cardio- and renoprotective effects of these drugs [118]. However, there is little evidence that treatment with ACEIs reduces CV morbidity and mortality in this particular population [12]. Furthermore, clinicians are often concerned about the possibly increased risk of adverse reactions from ACEI use in HF patients with impaired kidney function [118].

Experimental studies in animal models of uremia showed that ACEIs are able to prevent LVH and cardiomyocyte loss [119, 120], whereas administration of a bradykinin receptor inhibitor completely antagonizes these effects [120], suggesting that the beneficial effects of ACEIs on the CV system may be mediated through bradykinin.

Several observational studies [121] have suggested a favorable impact of ACEIs on survival in patients with CKD and HF. McAlister et al. [121] analyzed data from a prospective cohort of 754 patients with HF and found significant reductions in 1-year mortality with ACEIs and beta-blockers treatments in patients with eGFR <60 mL/min, as well as in those with eGFR ≥60 mL/min. A retrospective cohort study of 20,902 hospitalized elderly patients with a LVEF <40% [122] showed that, after adjustment for multiple confounders, the prescription of an ACEI on hospital discharge was associated with a significant reduction in mortality; notably, this reduction was greater in patients with serum creatinine >3 mg/dL (*n* = 1582) than in the rest of the cohort (37% versus 16%). Using propensity scores and multivariable-adjusted Cox regression analyses, Ahmed et al. [123] estimated the effect of ACEIs on 2-year outcomes in 1,707 patients with CKD, taken from the 6,800 patients with systolic HF (LVEF ≤45%) in the Digitalis Investigation Group trial. In this study, CKD was defined as serum creatinine ≥1.5 mg/dL for men and ≥1.3 mg/dL for women. Patients taking ACEIs had significantly lower rates of mortality (hazard ratio = 0.58) and all-cause hospitalizations (hazard ratio = 0.69), compared to those not taking ACEIs.

Moreover, benefits of ACEIs in patients with CKD and HF have been demonstrated by several* post hoc* analyses of RCTs conducted in the general HF population. The Survival and Ventricular Enlargement (SAVE) study was a randomized trial of captopril versus placebo in 2231 patients with acute myocardial infarction and LVEF ≤40%. Patients with serum creatinine <2.5 mg/dL were excluded. A secondary analysis of this trial showed that captopril was equally efficacious in subjects with CKD (defined as eGFR <60 mL/min/1.73 m^2^) and those without CKD. The relative risk reduction in CV events and mortality due to captopril was actually higher in subjects with CKD (31% versus 20%); however, the interaction between study drug and CKD was not statistically significant [124]. In the studies of left ventricular dysfunction (SOLVD) treatment trial, 2569 ambulatory chronic HF patients with LVEF ≤35% and serum creatinine ≤2.5 mg/dL were randomized to receive either placebo or enalapril. Of the 2502 patients with baseline serum creatinine data, 1036 had CKD (eGFR <60 mL/min/1.73 m^2^). The median follow-up was 35 months. Compared to placebo, enalapril significantly decreased all-cause mortality in non-CKD, but not in CKD, patients (hazard ratio 0.82 versus 0.88). However, enalapril did reduce CV hospitalization in both patients with and patients without CKD (hazard ratio 0.77 versus 0.80). Among patients in the enalapril group, serum creatinine elevation was significantly higher in those without CKD (0.09 versus 0.04 mg/dL) during the first year of follow-up, but there were no differences in changes in serum potassium (mean increase, 0.2 mEq/L, in both) [125].

In dialysis patients, observational studies have shown that ACEIs can reduce LVH [126, 127] and improve survival and CV outcomes [128], and these benefits appeared to be independent of their BP-lowering effect. However, a double-blind placebo-controlled RCT in 397 HD patients with LVH [129] failed to show any significant effect of ACEI fosinopril on a composite CV end-point. The study was, nevertheless, underpowered to estimate the impact of fosinopril on survival. Chang et al. evaluated the effects of ACEI use among HD patients that participated in the HEMO study [130]. Using proportional hazards regression and a propensity score analysis, the authors found no significant associations between ACEI use and mortality, CV hospitalization, and other CV outcomes. Surprisingly, in the proportional hazards model, ACEI use was even associated with a higher risk of HF hospitalization. A retrospective analysis of the data from the Minnesota Heart Survey [131] revealed that dialysis patients hospitalized with HF had no benefit from ACEI or ARB treatment, for either short-term (30 days) or long-term (1 year) survival, in striking contrast with all of the other HF patients.

Several concerns exist for the use of ACEIs and ARBs in patients with CKD, particularly about the risk of hyperkalemia and worsening of renal function. However, these effects are usually transient and mild.

A meta-analysis of 5 placebo-controlled RCTs with ACEIs in patients with HF showed that, although the rate of acute kidney injury was higher with ACEIs than with placebo, drug discontinuation was rarely necessary, and renal function returned to baseline in most cases, even without dose adjustment [118, 132]. Furthermore, a systematic review of 12 RCTs with ACEIs for renoprotection in patients with CKD showed that a mild increase in serum creatinine (up to 30% from baseline) was quite common within the first 2 weeks of therapy; however, this increase was followed by stabilization during the next few weeks [118, 133]. In patients with both HF and CKD, a retrospective analysis of the SOLVD studies has shown that the use of ACEIs was associated with a reduction of mortality, even in those with severe renal insufficiency, and did not have an adverse effect on kidney function [134]. Therefore, ACEIs should not be contraindicated in patients with HF and CKD, and a mild and nonprogressive worsening of renal function at the start of therapy should not be considered,* per se*, as an indication to discontinue treatment [118]. However, when the GFR falls by >30% of the pretreatment baseline, ACEI administration should be halted. Patients should then be evaluated for conditions causing renal hypoperfusion, such as volume depletion (e.g., from diuretics), renal vasoconstriction (e.g., induced by NSAIDs), and severe bilateral renal artery stenosis or stenosis in a single kidney. Unless renovascular disease is found, ACEI therapy can be resumed after correction of the underlying cause of renal ischemia and resolution of the acute kidney injury episode [118]. Reducing the daily diuretic and/or ACEI dose may prevent future worsening of the renal function [74]. It is generally recommended to begin at 15% to 25% of the goal dose and, based upon changes in BP and GFR, to increase every 4 to 8 weeks by 25% to 50% until the target dose or the highest tolerated dose is reached [135].

The risk of hyperkalemia associated with the use of ACEIs is also a source of concern. In a retrospective analysis of the SOLVD trials, in patients with HF treated with enalapril the incidence of hyperkalemia ≥5.5 mEq/L was 6%, overall; it was higher than in the placebo group and it increased progressively with the severity of the renal dysfunction [118, 136]. Careful monitoring of serum potassium is warranted in all patients with GFR <60 mL/min undergoing ACEI therapy. Concurrent use of other potentially hyperkalemia-inducing drugs, such as NSAIDs, ARBs, and potassium-sparing diuretics, should be avoided or minimized, if possible. A low potassium diet, as well as sodium bicarbonate administration in patients with metabolic acidosis, is also indicated [137]. A potassium level over 5.5 mEq/L should prompt a reduction in the ACEI dose. If the potassium concentration remains high despite the above measures, the ACEI should be discontinued [118, 137]. In patients with severe renal impairment, ACEIs should always be used with caution, because of their potential risk for adverse events.

#### 4.2.8. Angiotensin II Receptor Blockers

Experience with ARBs in HF trials is much smaller than that with ACEIs. However, several studies showed that ARBs produce hemodynamic, neurohormonal, and clinical effects similar to ACEIs. The ARBs valsartan and candesartan were associated with a reduction in hospitalizations and mortality in two HF RCTs [138, 139]. Given the existing evidence, ACEIs are currently recommended as the first choice for RAAS inhibition in HF, but ARBs are a reasonable alternative, especially for patients who cannot tolerate ACEIs because of cough or angioedema [32]. Side effects like hypotension, worsening renal function, and hyperkalemia are as common as for ACEIs. Therefore, caution is required, by starting treatment at very low doses, followed by slow, step-by-step increases. Additionally, BP, renal function, and serum potassium should be closely monitored.

The dual blockade of the RAAS for the treatment of HF, using a combination of an ACEI with an ARB, seems a reasonable approach. It was shown to reduce the LV size more than either agent alone [140]. However, the clinical benefits of this combination are uncertain. A trial in patients with HF postmyocardial infarction showed that combined therapy did not improve outcomes and resulted in more side effects, compared to each of the two drugs [141]. The addition of ARBs to chronic ACEI therapy caused a modest decrease in hospitalization in 2 studies, with a trend to decreased total mortality in one and no impact on mortality in another [32, 139, 140, 142]. Furthermore, the ACC/AHA guidelines suggest that this combination increases the risks of adverse effects [32].

In a study of patients with diabetic nephropathy and CKD stages 3-4, ARBs decreased the risk of developing HF [143]. In a* post hoc* analysis of the Telmisartan Randomized Assessment Study in ACE Intolerant Subjects with Cardiovascular Disease (TRANSCEND) and the Ongoing Telmisartan Alone and in Combination with Ramipril Global Endpoint Trial (ONTARGET), Tobe et al. [144] examined renal and CV outcomes in renal subgroups, defined by GFR and albuminuria. The main CV outcome was the composite of CV death, myocardial infarction, stroke, or hospitalization for HF. The authors found no CV benefit in any subgroup either with telmisartan versus placebo or with dual therapy (telmisartan plus ramipril) versus monotherapy.

Trials of ARBs in patients with HF and CKD are very scarce. In a recent cohort study of 1665 elderly patients with systolic HF (LVEF < 45%) and eGFR <60 mL/min/1.73 m^2^, followed up for 8 years, Ahmed et al. [145], using a propensity score analysis, found that treatment with ACEIs or ARBs was associated with a significant, but modest, reduction in all-cause mortality (hazard ratio 0.86; 95% confidence interval 0.74–0.996; *P* = 0.045) and no change in hospitalization for HF. A single RCT has been conducted so far using ARBs in ESRD patients. This multicenter Italian trial [146] included 332 HD patients with HF (NYHA II-III; LVEF ≤ 40%), who were randomized to telmisartan or placebo, in addition to ACEI therapy. At 3 years, telmisartan significantly reduced all-cause mortality (35.1% versus 54.4%; *P* < 0.001), CV death (30.3% versus 43.7%; *P* < 0.001), and hospital admission for HF (33.9% versus 55.1%; *P* < 0.0001). Adverse effects, mainly hypotension, occurred in 16.3% of the telmisartan group versus 10.7% in the placebo group.

#### 4.2.9. Aldosterone Antagonists

RAAS inhibition with ACEIs and/or ARBs may not be able to maintain adequate suppression of aldosterone production during long-term therapy, because both aldosterone and angiotensin II ultimately can escape the effects of these drugs, resulting in rebound of aldosterone levels [147, 148]. This may be a significant issue in patients with HF, since experimental studies suggest that aldosterone has deleterious effects on the structure and function of the heart, independently of and in addition to those of angiotensin II [32]. Aldosterone stimulates sodium and fluid retention and promotes myocardial remodeling and fibrosis, as well as endothelial dysfunction and atherosclerosis [149, 150]. Aldosterone antagonists (AAs), in addition to ACEIs or ARBs, can provide more complete inhibition of the RAAS, with long-term benefits. However, a higher risk of adverse effects like hyperkalemia and worsening renal function is also to be expected.

Spironolactone and eplerenone were associated with significant reductions in mortality and CV events in patients with systolic HF in the RALES (Randomized Aldactone Evaluation Study) [151] and EPHESUS (Eplerenone Post-Acute Myocardial Infarction Heart Failure Efficacy and Survival) trials [152, 153], respectively. On the other hand, many studies have reported an increased incidence of severe hyperkalemia in HF patients treated with AAs in association with ACEIs [118]. Based on these data, US [32] and European [30] guidelines recommend the addition of an AA to an ACEI or an ARB in selected patients with systolic HF (NYHA classes III-IV, LVEF <35%), but without severe renal dysfunction (serum creatinine ≤ 2.5 mg/dL in men and ≤ 2.0/dL in women) and with serum potassium <5.0 mEq/L. Treatment should be initiated at low doses (e.g., 12.5 or 25 mg of spironolactone or eplerenone), followed by a gradual increase (up to a target of 50 mg, if tolerated), under careful surveillance of creatinine and potassium levels. Hyperkalemia and/or worsening of the renal function require dose reduction or even withdrawal of AAs. In men, breast tenderness or enlargement may also occur with spironolactone therapy, in which case switching to eplerenone is indicated. The use of AAs should be avoided whenever adequate monitoring of potassium and creatinine levels is deemed not feasible. Furthermore, AAs are contraindicated in association with other potassium-sparing diuretics, with potassium supplements, and with combinations of ACEIs and ARBs [30, 32].

The effects of AAs on clinical outcomes in patients with HF and moderate or severe CKD are not clear, since both RALES and EPHESUS trials excluded patients with serum creatinine levels >2.5 mg/dL. A prospective RCT in 112 patients with stages 2 and 3 CKD showed a significant improvement in LV mass and arterial stiffness with spironolactone versus placebo, independently of central and peripheral BP changes [154]. In Iran, Taheri et al. conducted a small double-blind RCT of spironolactone 25 mg/day versus placebo, in addition to an ACEI or an ARB, in 16 HD patients with HF (NYHA classes III-IV and LVEF < 45%). After 6 months of treatment, the mean LVEF increased significantly more in the spironolactone group than in the placebo group and the mean LV mass decreased in the spironolactone group, while it increased significantly in the placebo group. The incidence of hyperkalemia was unchanged in both groups [155]. The same research team performed a study with an identical design in 18 PD patients with HF. They found a significant increase in LVEF in the spironolactone group but not in the placebo group and a nonsignificant increase in serum potassium in both groups [156].

The risk of AA-induced hyperkalemia in patients with advanced CKD has rarely been assessed in prospective studies, but most experts believe that this risk is unacceptably high and may become life-threatening, therefore prohibiting the use of these drugs in patients with severe and end-stage kidney disease. However, it has been suggested that hyperkalemia may be a less serious issue in HD patients, due to the effective removal of potassium through dialysis, as well as to the ability of these patients to tolerate relatively high levels of potassium without clinical manifestations [157]. Chua et al. recently reviewed 6 RCTs that evaluated the safety of low-dose spironolactone in HD patients (of which, about 50% were already on ACEI or ARB therapy). The authors found that the incidence of hyperkalemia with spironolactone treatment was similar to that in control groups; however, all these studies involved small populations of compliant subjects, who were at low risk for hyperkalemia [157].

Large-scale RCTs are required to evaluate the efficacy and safety of AAs in addition to ACEIs or ARBs as a treatment strategy for HF in CKD patients. In stage 3 CKD patients with HF, AAs may be considered but should be used with great caution, limiting the dose to 25 mg/day, or every other day, and closely monitoring the potassium levels. The AAs should be avoided in patients with CKD stages 4 and 5 [118], although potassium removal by dialysis may lessen the risk of hyperkalemia in patients on renal replacement therapy. The combined use of all three RAAS inhibitors (ACEIs, ARBs, and AAs) cannot be recommended in HF patients, with or without CKD [32].

#### 4.2.10. Digitalis Glycosides

In HF, digitalis glycosides act by inhibiting the Na-K-ATP-ase in the myocardium, thereby increasing cardiac contractility. More recently, these drugs were shown to inhibit Na-K-ATP-ase in noncardiac tissues, as well. In the vagal fibers, this enzymatic inhibition results in a decrease of central nervous system sympathetic activity, whereas in the kidneys it leads to a reduction of renal sodium reabsorption, with subsequent suppression of the RAAS. It has in fact been suggested that these neurohormonal effects of digitalis may be even more important than its myocardial inotropic effect, in terms of clinical benefits for patients with HF [32].

Several small RCTs have shown that short-term treatment with digoxin can improve symptoms, quality of life, and exercise tolerance in patients with HF. These favorable outcomes have been seen both in patients with normal sinus rhythm and in those with atrial fibrillation. In the randomized placebo-controlled Digitalis Investigation Group (DIG) trial [158], in 6800 HF patients with LVEF ≤45% and NYHA classes II–IV, already receiving an ACEI, treatment with digoxin for 2 to 5 years reduced hospitalizations but had no effect on overall mortality. The outcomes did not vary significantly in relation to baseline GFR [159]. However, a* post hoc* analysis of this trial [160] showed that the effects of digoxin on mortality were dependent on its serum concentrations: at 0.5 to 0.9 ng/mL, digoxin significantly reduced mortality in all HF patients, including those with preserved systolic function, whereas, at higher concentrations, it had no effect in this regard. Low serum digoxin concentration was associated with reduced mortality in most subgroups, including CKD (GFR < 60 mL/min/1.73 m^2^). In the DIG trial, however, individuals with creatinine levels >3.0 mg/dL were not eligible, and only 3% of participants had a GFR <30 mL/min/1.73 m^2^ [159].

US and European guidelines recommend considering the addition of digoxin in patients with HF who are already treated with optimal doses of diuretics, ACEIs (or ARBs), and beta-blockers and who still have symptoms of HF. Alternatively, an AA could be tried in such cases and digoxin would further be indicated only for those who do not respond or who cannot tolerate AAs. In patients with symptomatic HF and atrial fibrillation, digoxin can be used to control the heart rate, in addition to, or prior to, a beta-blocker. A daily dose of digoxin of 0.25 mg is most often employed in patients with normal renal function. Potential adverse effects of digitalis include sinoatrial and atrioventricular blocks, atrial and ventricular arrhythmias (especially in the presence of hypokalemia), confusion, nausea, and disturbance of color vision. Digoxin is contraindicated in patients with second- or third-degree heart blocks, suspected sick sinus syndrome, and preexcitation syndromes [32].

Because 85% of administered digoxin is excreted by the kidneys, the risk of toxicity from this drug is very high in people with CKD [74]. Considering the narrow therapeutic window, the long half-life, and the potential for lethal arrhythmias (especially in the context of HD-induced hypokalemia), most nephrologists generally avoid using digoxin in patients with advanced CKD and ESRD [161]. Chan et al. analyzed the association between digoxin prescription and survival in a retrospective cohort, using covariate- and propensity-score-adjusted Cox models. In over 120,000 incident HD patients, digoxin use was associated with a 28% increased risk for death. Increasing serum digoxin concentration was also significantly associated with mortality, most markedly in patients with lower predialysis serum potassium [162].

#### 4.2.11. Cardiac Resynchronization Therapy

Dyssynchronous ventricular contraction is often seen in patients with HF and it is usually recognized by the presence of a wide QRS >0.12 s on electrocardiogram. Ventricular dyssynchrony results in deficient LV filling, slower rate of increase in LV contractile force, significant mitral regurgitation, paradoxical septal wall motion, and reduced cardiac output [32, 163] and has been associated with increased mortality in patients with systolic HF [164, 165]. Cardiac resynchronization therapy (CRT) employs a biventricular pacemaker device that electrically activates the right and left ventricles in a synchronized manner. Such devices often provide a defibrillator function, as well. Resynchronization therapy can improve ventricular contraction and reduce mitral regurgitation. In a meta-analysis of 14 RCTs that included a total of 4420 patients with moderate or severe HF, systolic dysfunction, and prolonged QRS, in addition to medical treatment, CRT significantly improved LVEF, quality of life, and functional status and decreased hospitalizations by 37% and all-cause mortality by 22% [166]. Based on these trials, CRT with defibrillator function is currently recommended for HF patients in NYHA III-IV classes who are symptomatic despite optimal medical therapy and who have a LVEF ≤35% and QRS prolongation >0.12 s [30, 32]. Complications of CRT include lead malfunction or dislodgement, pacemaker problems, and infection.

Most RCTs with resynchronization devices in HF populations had little data on patients with CKD [167]. However, renal subgroup analyses of some of these trials have revealed that the clinical benefits of CRT were similar in all studied patients, irrespective of their baseline GFR. For example, the Multicenter InSync Randomized Clinical Evaluation (MIRACLE) study evaluated CRT in HF patients with NYHA classes III-IV, LVEF ≤35%, and QRS >0.12 s, but with serum creatinine ≤3.0 mg/dL. A retrospective analysis of this trial [168] categorized patients according to their baseline eGFR: ≥90 (A), 89 to 60 (B), and 59 to 30 (C) mL/min/1.73 m^2^. The authors found that, compared to control, CRT improved NYHA class, increased LVEF, and reduced mitral regurgitation in all three eGFR categories, while it improved exercise capacity and decreased LV mass in category C. Furthermore, CRT significantly increased eGFR in category C, suggesting that CRT can indirectly improve renal function, by improving the cardiac function.

Considering the very limited available data, it is difficult to make evidence-based recommendations regarding the use of CRT in CKD patients. Adequate studies are required to evaluate the effect of CRT on morbidity and mortality in this specific population. In HD patients, the transvenous placement of CRT and other cardiac rhythm devices has been associated with an increased risk of device-related infections and central vein stenosis. To avoid such risks, the use of an epicardial approach has recently been suggested for CRT devices in these patients, rather than the classical transvenous route [169].

#### 4.2.12. Optimization of Dialysis

Adequate ultrafiltration is a useful strategy for controlling overhydration and hypertension in dialysis patients. Dietary sodium restriction and the use of low dialysate sodium concentrations are equally important in this regard [12].

In non-ESRD patients with severe, refractory HF, several small studies have demonstrated that ultrafiltration (using either HD or PD techniques) can reduce volume overload, correct hyponatremia, and restore responsiveness to diuretics [30, 32]. In patients with ESRD and HF, the initiation of dialysis may have favorable effects on the heart structure, possibly due to decrease of fluid overload. In a recent retrospective echocardiographic study of 41 patients with advanced CKD and symptomatic HF with low LVEF, Ganda et al. [170] found a significant reduction in LV mass index within a few months from the start of HD, although there was no change in LV shortening fraction.

It has also been suggested that HD might improve cardiac and vascular structure and function in patients with HF, by removal of inflammatory cytokines such as interleukin-8 and monocyte chemotactic protein-1 [171]. However, the true therapeutic role of this process is unclear, considering that the mass clearance of these cytokines is rather low, that they have a short half-life and can rapidly reappear in plasma, and that beneficial cytokines are lost along with the potentially harmful ones, while the source of inflammation is not affected [89].

On the other hand, HD sessions are often associated with repetitive hemodynamic instability and subsequent myocardial ischemia, resulting in “myocardial stunning” [172]. These short episodes may lead over time to development of permanent regional LV systolic abnormalities and HF [173]. Avoiding large-volume ultrafiltration [69] and improving intradialytic hemodynamics by use of biofeedback mechanisms and cooled dialysate [174, 175] might reduce the incidence of such myocardial stunning events. High-flow arteriovenous fistula should also be avoided, as it was shown to contribute to volume overload, high cardiac output, and eccentric LVH [176].

Several studies have suggested that more intensive HD (e.g., short daily or long nocturnal dialysis) has significant CV benefit, compared to conventional thrice-weekly HD [69]. Charra et al. [177] were the first to show that prolonged HD (thrice weekly, 8 hours per session) led to a progressive decrease in volume overload and BP. In a small short-term randomized trial, Culleton et al. [178] showed marked reductions in LVH and systolic BP in patients using frequent nocturnal HD (i.e., 6–8 hours of dialysis treatment at home for 4–6 nights per week), compared to those on conventional HD. Similar results were reported by Ayus et al. [179] in a nonrandomized prospective cohort study of short daily HD versus conventional HD. Chan et al. [180] showed that conversion from conventional to frequent nocturnal HD was associated with a decrease in 24-hour mean BP, peripheral vascular resistance, sympathetic activity, arterial stiffness, and LV mass. In another study, the same authors observed a significant improvement in LVEF in dialysis patients with LV systolic dysfunction, after switching from conventional to frequent nocturnal HD [181]. Finally, the CV benefits of intensive HD have been confirmed by two very recent RCTs, which compared conventional HD to 6 times per week daily in-center HD (the Frequent Hemodialysis Network Daily Trial) and to 6 times per week nocturnal HD (the FHN Nocturnal Trial), respectively. In the Daily Trial, frequent HD resulted in a significant reduction in LV mass (especially in patients with baseline LVH), whereas similar trends were noted in the Nocturnal Trial [182]. All of the above data suggest that ESRD patients with HF might derive substantial benefits from using such intensive HD programs rather than conventional ones. This hypothesis should be considered for further trials.

There is evidence that, during the first few years of dialysis therapy, while residual renal function is preserved, PD may provide better fluid and BP control than HD [183–185], probably due to more abundant urine output and continuous, slow ultrafiltration. However, the prevalence of hypertension and overhydration increases after several years on PD, as a result of progressive loss of diuresis and of peritoneal ultrafiltration capacity [186–189]. Peritoneal ultrafiltration failure is associated with volume expansion, hypertension, LVH, and inflammation [190]. Nevertheless, the use of hypertonic PD solutions, together with salt intake restriction, can maintain adequate BP control and prevent LVH, despite the reduction of residual renal function in long-term PD patients [191]. Icodextrin-based PD solution is a promising alternative, as it was shown to significantly reduce volume overload and LV mass, compared with a standard solution [192]. Whether this translates to a long-term survival benefit in PD patients with HF warrants further investigation.

To date, there are no RCTs comparing the efficacy of PD versus HD for the management of dialysis patients with HF. In a cohort of over 100,000 incident dialysis patients with a history of HF, Stack et al. [193] found that PD was associated with a significantly higher mortality, compared with HD, after 2 years of therapy. On the other hand, Vonesh et al. [194] examined data of almost 400,000 dialysis patients (11.6% on PD), followed from dialysis inception up to 3 years, and found that those with baseline HF had similar survival rates on both modalities, except for the subgroup of diabetics aged over 45 years, which had a lower mortality on HD than on PD.

#### 4.2.13. Future Directions

Direct renin inhibitors (DRIs) are a newer class of RAAS inhibitors, acting at the first regulatory step of this hormonal system. Initially used as antihypertensive agents, DRIs have more recently been tested in patients with HF. In the ALOFT (Aliskiren Observation of Heart Failure Treatment) study, which included 302 patients with stable HF, adding the DRI aliskiren to ACEIs or ARBs appeared to be safe and effective in decreasing plasma BNP and urinary aldosterone levels [195]. Two other large trials are underway using aliskiren in HF patients. The ATMOSPHERE (Aliskiren Trial to Minimize Outcomes in Patients with Heart Failure) examines the effect of aliskiren on CV mortality and hospitalization in patients with chronic HF, whereas the ASTRONAUT (Aliskiren Trial on Acute Heart Failure Outcomes) evaluates aliskiren in patients stabilized after acute HF [196]. These studies will shed important light on the role of DRIs in the treatment of HF. However, we should mention here the Aliskiren Trial in Type 2 Diabetes Using Cardio-Renal Disease Endpoints (ALTITUDE), which compared aliskiren to placebo, in addition to ACEI or ARB therapy in patients with diabetic nephropathy and CV disease. This study was prematurely stopped, because of the lack of any prospects of showing a treatment benefit, as well as due to safety concerns, including renal dysfunction, hyperkalemia, hypotension, and an unexpected excess of strokes. As a consequence, it has been suggested that dual aliskiren and ACEI/ARB therapy should not be used in patients with hypertension and CKD (eGFR <60 mL/min/1.73 m^2^) [195].

BAY 94-8862 is a novel, nonsteroidal, mineralocorticoid receptor antagonist with greater selectivity than spironolactone and stronger binding affinity than eplerenone. The MinerAlocorticoid Receptor Antagonist Tolerability Study (ARTS) was a multicentre, randomized, double-blind, placebo-controlled, parallel-group study, aiming to evaluate the safety and tolerability of this new drug in patients with systolic HF and CKD [197]. This study showed that BAY 94-8862 5–10 mg/day was at least as effective as spironolactone 25 or 50 mg/day in decreasing serum levels of BNP and pro-BNP, as well as albuminuria, but it was associated with lower incidence of hyperkalaemia (5.3% versus 12.7%; *P* = 0.048).

Other innovative therapies have been explored in the general population with HF, but with unconvincing results so far. These include TNF-*α* inhibitors etanercept and infliximab [198, 199], the endothelin antagonist bosentan [200], the combined ACE and neutral endopeptidase inhibitor omapatrilat [201], and tolvaptan, a vasopressin (V2) antagonist [202]. Drugs that reverse myocardial fibrosis and matrix remodeling by antagonizing the TGF-*β* pathway or by blockade of further downstream pathways also are under investigation [203]. Further research is needed to clarify the role of these agents in HF patients, including those with CKD.

## 5. Conclusions

1. Dietary salt restriction and diuretics are recommended for patients with CKD and HF to control fluid overload and symptoms, although their effects on morbidity and mortality are unknown. Loop diuretics should be used as first-line agents in patients with GFR <30 mL/min/1.73 m^2^.
2. In patients with CKD and ESRD, anemia has been associated with LV hypertrophy and dilatation. In the absence of specific RCTs, we suggest that in people with CKD and HF anemia should be treated according to the guidelines used in the general CKD population, targeting a serum hemoglobin of 11 to 12 g/dL.
3. Hyperphosphatemia, secondary hyperparathyroidism, and vitamin D deficiency have all been associated with LV hypertrophy and dysfunction. Achieving adequate phosphate, calcium, vitamin D, and PTH levels is a reasonable treatment goal in CKD patients, with or without heart disease, although their benefits for preventing or improving HF in these patients have not been proven so far.
4. In CKD (as well as in non-CKD) patients with systolic HF, several RCTs have shown that beta-blockers (bisoprolol, metoprolol, and carvedilol) can reduce mortality and hospitalization rates. Therefore, a beta-blocker should be recommended to all such patients, unless contraindicated or not tolerated. Treatment must be started at a very low dose and carefully uptitrated and monitored, in order to avoid worsening HF, bradycardia, and hypotension (particularly in dialysis patients). The role of beta-blockers in patients with CKD and HF with normal LVEF is unknown.
5. Several* post hoc* analyses of RCTs conducted in the general HF population have shown a favorable effect of ACEIs on survival in patients with CKD and HF; however, subjects with serum creatinine >2.5 mg/dL were excluded from those trials. Considering the well-known CV and renal benefits, we believe that ACEIs should be indicated to all patients with HF and mild-to-moderate CKD (stages 1–3), unless contraindicated or not tolerated. On the other hand, in people with HF and advanced CKD or ESRD, the benefits of ACEIs on survival have not been proven and, furthermore, there is a higher risk of adverse events. Therefore, caution is required in patients with HF and severely impaired renal function (CKD stages 4 and 5). As an alternative to ACEIs, ARBs can be used, particularly in patients who develop cough or angioedema from ACEIs. Dual therapy with ACEIs and ARBs can also be considered, especially in resistant cases, although the advantage over monotherapy is still uncertain and the risk of adverse effects is likely increased. When using RAAS inhibitors, careful dose titration and clinical monitoring are required to prevent serious side effects, such as hypotension, hyperkalemia, and acute kidney injury. The role of ACEIs and ARBs in patients with CKD and HF with normal LVEF is unknown. In stage 3 CKD patients with HF, aldosterone antagonists may be considered but should be used with great caution and at very low doses, while closely monitoring the potassium levels. Aldosterone antagonists should be avoided in patients with CKD stages 4 and 5.
6. The use of digoxin has very limited indications and requires great prudence in patients with HF and CKD. The administration of this drug may be considered in selected cases with poorly controlled symptoms of HF or with high-ventricular rate atrial fibrillation, in the presence of optimal-dose therapy with diuretics, RAAS inhibitors, and beta-blockers. Monitoring of serum digoxin concentration is required, with a target of 0.5 to 0.9 ng/mL; this is usually achieved by administering the drug at very low doses (e.g., 0.125 mg every other day when GFR is 30 to 60 mL/min and less frequently in patients with more severe renal dysfunction).
7. Considering the very limited evidence, no recommendations can be made regarding cardiac resynchronization therapy in CKD patients with HF.
8. For ESRD patients with HF, the role of dialysis modality choice (HD versus PD) is unclear, but likely irrelevant. However, in both HD and PD patients, adequate ultrafiltration is crucial for controlling volume overload. In HD, large-volume ultrafiltration should be avoided, as it may be associated with myocardial stunning episodes. High-flow arteriovenous fistula should also be avoided, as it could contribute to volume overload, high cardiac output, and eccentric LVH. Intensive HD schedules (e.g., short daily or long nocturnal dialysis) and the use of icodextrin-based PD solutions may result in better fluid status and LVH reduction, but whether this translates into survival benefits for HF patients is still unknown.

These conclusions are summarized in Box 1.

## Supplementary Material

Table S1: Medline and Cochrane search strategy.

## Figures and Tables

**Box 1:**
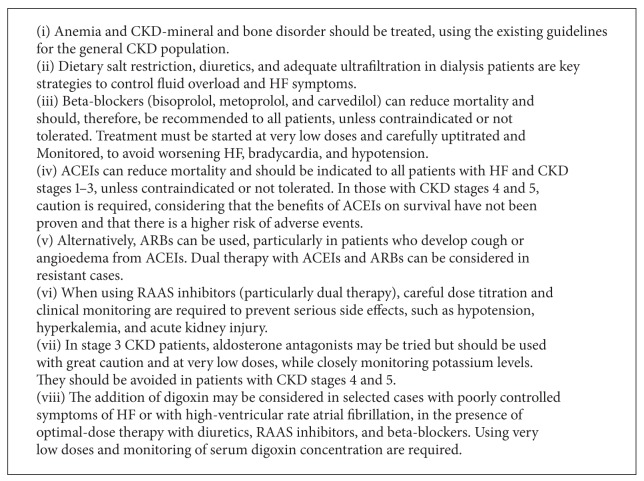
Treatment of HF in patients with CKD: key messages.
