# Supplementary material for: Heart Failure in Patients with Chronic Kidney Disease: A Systematic Integrative Review
Source: Biomed Res Int. 2014 May 15;2014:937398. doi: 10.1155/2014/937398 (PMC4052068; doi:10.1155/2014/937398)
Supplement: Supplementary file 1 — Table S1: Medline and Cochrane search strategy. [file 937398.f1.docx]

Table S1 Search Strategies

| Database | Search terms used |  |
| --- | --- | --- |
| Medline | 1. Kidney Diseases/  2. exp Renal Replacement Therapy/  3. Renal Insufficiency/  4. exp Renal Insufficiency, Chronic/  5. dialysis.tw.  6. (hemodialysis or haemodialysis).tw.  7. (hemofiltration or haemofiltration).tw.  8. (hemodiafiltration or haemodiafiltration).tw.  9. (end-stage renal or end-stage kidney or endstage renal or endstage kidney).tw.  10. (ESRF or ESKF or ESRD or ESKD).tw.  11. (chronic kidney or chronic renal).tw.  12. (CKF or CKD or CRF or CRD).tw.  13. (CAPD or CCPD or APD).tw.  14. (predialysis or pre-dialysis).tw.  15. or/1-14  16. exp Heart Failure/  17. (heart adj2 failure*).tw.  18. (congestive adj2 heart).tw.  19. (cardiac adj2 failure*).tw.  20. (heart adj2 decompensation*).tw.  21. (myocardial adj2 failure*).tw.  22. paroxysmal dyspnea*.tw.  23. cardiac asthma.tw.  24. cardiac edema*.tw.  25. or/16-24  26. 15 and 25 |  |
| CENTRAL | #1 dialysis:ti,ab,kw  #2 h*emofiltration:ti,ab,kw  #3 h*emodiafiltration:ti,ab,kw  #4 (end-stage renal or end-stage kidney or endstage renal or endstage kidney):ti,ab,kw  #5 (ESRF or ESKF or ESRD or ESKD):ti,ab,kw  #6 (chronic kidney or chronic renal):ti,ab,kw  #7 (CKF or CKD or CRF or CRD):ti,ab,kw  #8 (CAPD or CCPD or APD):ti,ab,kw  #9 (predialysis or pre-dialysis):ti,ab,kw  #10 MeSH descriptor: [Kidney Failure, Chronic] this term only  #11 MeSH descriptor: [Renal Replacement Therapy] explode all trees  #12 MeSH descriptor: [Renal Insufficiency, Chronic] explode all trees  #13 #1 or #2 or #3 or #4 or #5 or #6 or #7 or #8 or #9 or #10 or #11 or #12  #14 MeSH descriptor Heart Failure explode all trees  #15 heart near/6 failure*  #16 congestive near/6 heart  #17 cardiac near/6 failure*  #18 heart near/2 decompensation  #19 myocardial near/6 failure*  #20 paroxysmal next dyspnea*  #21 cardiac next asthma  #22 cardiac next edema*  #23 (#14 or #15 or #16 or # 17 or #18 or #19 or #20 or #21 or #22)  #24 #13 and #23 |  |
